# Post-transcriptional regulation of ribosome biogenesis in yeast

**DOI:** 10.15698/mic2017.05.575

**Published:** 2017-05-01

**Authors:** Isabelle C. Kos-Braun, Martin Koš

**Affiliations:** 1 Biochemistry Center, University of Heidelberg, Im Neuenheimer Feld 328, 69120 Heidelberg, Germany.

**Keywords:** Tor pathway, TORC1, Casein kinase 2 (CK2), rRNA processing, ribosome biogenesis, yeast

## Abstract

Most microorganisms are exposed to the constantly and often rapidly changing environment. As such they evolved mechanisms to balance their metabolism and energy expenditure with the resources available to them. When re-sources become scarce or conditions turn out to be unfavourable for growth, cells reduce their metabolism and energy usage to survive. One of the major energy consuming processes in the cell is ribosome biogenesis. Unsurprisingly, cells encountering adverse conditions immediately shut down production of new ribosomes. It is well established that nutrient depletion leads to a rapid repression of transcription of the genes encoding ribosomal proteins, ribosome biogenesis factors as well as ribosomal RNA (rRNA). However, if pre-rRNA processing and ribosome assembly are regulated post-transcriptionally remains largely unclear. We have recently uncovered that the yeast *Saccharomyces cerevisiae* rapidly switches between two alternative pre-rRNA processing pathways depending on the environmental conditions. Our findings reveal a new level of complexity in the regulation of ribosome biogenesis.

The process of ribosome synthesis is conserved from prokaryotes to eukaryotes. It starts by the transcription of a large pre-rRNA that is subsequently processed into mature rRNAs and assembled with ribosomal proteins into the ribosomal subunits. During the processing, the pre-rRNA is cleaved at several positions, trimmed by exonucleases and covalently modified at a number of sites. The making of ribosomes in eukaryotes requires a coordinated action of all three RNA polymerases, dozens of small nucleolar RNAs (snoRNAs) and hundreds of proteins - accessory ribosome biogenesis factors and ~80 ribosomal proteins. It was estimated that in a fast growing yeast cell up to 60% of all transcription represents transcription of the ribosomal RNA (rRNA) by RNA polymerase I. In addition, transcription of the ribosomal protein genes accounts for 50% of the RNA polymerase II activity. Furthermore, the RNA polymerase III synthesises the 5S rRNA. The Tor complex 1 (TORC1) pathway controls the activity of all three RNA polymerases in concordance with the nutrient availability. When nutrients are abundant, the Tor1 kinase is active and promotes high levels of transcription. A depletion of nutrients leads to inactivation of TORC1 and cessation of the transcription of rDNA and ribosomal protein genes.

We recently observed that nutrient depletion leads to a striking change in the pre-rRNA processing pattern. We showed that yeast switches promptly between the two alternative ribosome biogenesis pathways in response to nutrients availability and certain environmental stresses. We named the two alternative pre-rRNA processing pathways after the specific cleavage site that distinguishes them as A2-pathway and A3-pathway, respectively. Under favourable growth conditions, the pre-rRNA is cleaved at the site A2 to split the large precursor into 20S and 27S-A2 pre-rRNAs (A2-pathway). In contrast, under unfavourable growth conditions, the pre-rRNA is cleaved at the site A3 yielding the 23S and 27S-A3 pre-rRNAs (A3-pathway). In agreement with previous observations, the transcription of rDNA also decreases. However, we found that the synthesis of pre-rRNA, while strongly reduced, continues for days but the produced pre-rRNA is cleaved differently. We estimate that the rDNA transcription is reduced to ~2% after the depletion of glucose. This still represents a significant amount of transcription, as rDNA is very highly transcribed - each active rDNA unit can be transcribed simultaneously by 50- 100 RNA polymerases I and there are 75-100 active rDNA units in yeast. Significantly, we could not detect any new ribosomes being made in our *in vivo* pulse-chase experiments, indicating that the A3-pathway is not productive. The Northern blot analyses showed that the pre-rRNA processing and assembly of both ribosomal subunits is stalled after the first cleavages. While in favourable conditions the 20S and 27S-A2 pre-rRNA (produced by a cleavage at the site A2) are rapidly matured into ribosomal subunits, the 23S pre-rRNA is not further processed and the synthesis of the small ribosomal subunit seems to be stalled at this step. Similarly, the 27S-A3 is only quickly converted to 27S-B pre-rRNA and the processing also stalls at this point and no new large ribosomal subunits are produced (Figure 1).

**Figure 1:**
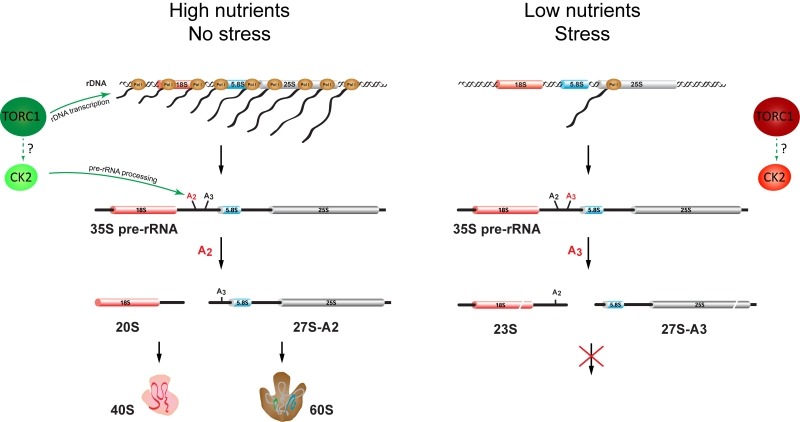
Figure 1: Alternative pathways of the pre-rRNA processing in yeast. Left: When nutrients are available and no environmental stress is present, TORC1 and CK2 kinase are active, promoting high level of rDNA transcription, cleavage of the pre-rRNA at the site A2 and maturation of new ribosomes. Right: In the condition with low nutrients or stress, both TORC1 and CK2 are inactive. The transcription of rDNA is reduced and pre-rRNA is cleaved at the site A3 and no new ribosomes are produced.

Interestingly, the existence of the 23S pre-rRNA was reported two decades ago but its functional relevance remained highly controversial. As the 23S is readily detectable in the yeast strains with mutations affecting ribosome biogenesis, it has been mostly dismissed as a product of aberrant processing. Our results show that it is a physiologically relevant intermediate produced under unfavourable conditions. The meaning of the 23S appearance in the mutant strains needs to be critically re-evaluated as it might represent a general stress response to defects in ribosome biogenesis rather than a direct consequence of the particular mutation in the yeast strain.

The nutrients availability and stress sensing in yeast is mediated by the four kinases Tor1, PKA, Snf1 and Pho85 via their signalling cascades. We found that the switch between the alternative pre-rRNA processing pathways is controlled by the Tor1 kinase, or more specifically the TORC1. Inactivation of TORC1 by rapamycin caused a rapid switch from A2- to A3-pathway. The TORC1 signalling pathway has two major branches, one controlled by the Sch9 kinase (the yeast homologue of the mammalian S6 kinase) and the other controlled by the Tap42 kinase. The Sch9 kinase regulates all three RNA polymerases, directly controlling the synthesis of the pre-rRNA and mRNAs for ribosomal proteins and ribosome biogenesis factors (RiBi genes). Surprisingly, we discovered that both Sch9 and Tap42 functions are dispensable for the switch in pre-rRNA processing, indicating that TORC1 has another so far unrecognized or unidentified effector regulating directly the pre-rRNA processing. We therefore looked at the casein kinase 2 (CK2), which is a component of the early pre-ribosomes. Importantly, the CK2 kinase is also a component of the CURI complex consisting of CK2, Utp22, Rrp7 and Ifh1. Whereas the Utp22 and Rrp7 are essential bona-fide ribosome biogenesis factors, the Ifh1 is a transcriptional coactivator required for the efficient transcription of ribosomal protein genes. Therefore, the CURI complex was therefore proposed to link ribosome biogenesis with the expression of ribosomal proteins. Thus CK2 represents are good candidate for direct regulation of the pre-rRNA processing. We showed that inhibition of the CK2 activity resulted in a fast switch from A2- to A3-pathway. Interestingly, small amounts of 27S-A2 pre-rRNA (produced by the A2-pathway) were still detectable at the initial time point after CK2 inhibition indicating that CK2 kinase activity is required also for the further downstream processing of the 27S-A2 precursor. Our proteomic analyses of pre-ribosomes identified a number of proteins with changes in the phosphorylation at various sites, including potential CK2 target sites. Further investigation of the identified candidates is needed to gain insight into the link between TORC1 and CK2.

Our observations raise many important questions regarding the underlying mechanism of the switch. Can the switch in the pre-rRNA processing be explained without the need to invoke a new target of TORC1? The transcription of rDNA and expression of ribosomal proteins are reduced strongly after nutrient depletion. Is it the smaller number of RNA polymerases I or their speed with which they are passing through rDNA together with the changed availability of ribosomal proteins that affect co-transcriptional folding of the pre-rRNA to physically block the processing at the A2 site? We addressed these possibilities by several experiments. First, we showed that the switch in processing occurs regardless whether the rDNA is transcribed by RNA polymerase I or II. Second, acute sequestering of the catalytic subunit of RNA polymerase I by the anchor-away technique, which effectively reduced the number of available RNA polymerases I, did not elicit the switch from A2 to A3 pathway. Third, using the strain with RNA polymerase I insensitive to rapamycin we showed that sustained transcription of rDNA does not prevent switch to A3 pathway. We also showed that the content of ribosomal proteins in the pre-ribosomes produced by the A3-pathway is not changed or even increased compared to A2-pathway pre-ribosomes. This demonstrates that a general lack of ribosomal proteins after reduction of their expression is also unlikely the cause of the change in the pre-rRNA processing. All these observations together with the rapidity of the switch point to a direct control of the pre-rRNA processing. Nevertheless, it is not clear if there is indeed just one target protein that is fully responsible for the regulation of the cleavage site choice. Pre-ribosomes are immensely dynamic and complex machines. It is plausible that subtle rearrangements of the pre-ribosomes, driven by post-translational protein modifications, affect the choice of the cleavage site.

Finally, what is the advantage for yeast to switch to a non-productive pre-rRNA processing? One explanation could be an evolutionary advantage increasing the competitive fitness of the organism. A complete abolishment of rDNA transcription causes dissolution of the nucleoli, the ribosome factories, with ribosome biogenesis factors diffusing away. Restarting the ribosome production with an "empty factory" will take significantly longer than with a factory still running at minimal level. In nature, even a small delay can lose the battle with other faster species.

Our findings reveal a new mode of regulation of ribosome biogenesis. Intriguingly, different cleavage sites can also be used in the human pre-rRNA processing. Whether a mechanism similar to yeast is conserved and used by cells depending on their proliferation status remains to be seen. As a dysregulated ribosome biogenesis is observed in many cancers, a better understanding of its regulation might have far reaching implications also for human health.

